# Multicenter Validation Study of the American Joint Commission on Cancer (8th Edition) for Gastric Cancer: Proposal for a Simplified and Improved TNM Staging System

**DOI:** 10.7150/jca.36891

**Published:** 2020-03-13

**Authors:** Jian-Xian Lin, Jacopo Desiderio, Jun-Peng Lin, Wei Wang, Ru-Hong Tu, Ping Li, Jian-Wei Xie, Jia-Bin Wang, Jun Lu, Qi-Yue Chen, Long-Long Cao, Mi Lin, Chao-Hui Zheng, Zhi-wei Zhou, Amilcare Parisi, Chang-Ming Huang

**Affiliations:** 1 Department of Gastric Surgery, Fujian Medical University Union Hospital, Fuzhou, China; 2 Key Laboratory of Ministry of Education of Gastrointestinal Cancer, Fujian Medical University, Fuzhou, China; 3 Department of Digestive Surgery, St. Mary's Hospital, University of Perugia, Terni, Italy; 4 Department of Gastric and Pancreatic Surgery, Sun Yat-sen University Cancer Center, State Key Laboratory of Oncology in South China, Collaborative Innovation Center for Cancer Medicine, Guangzhou, China

**Keywords:** gastric cancer, radical gastrectomy, TNM classification, prognosis

## Abstract

**Objective:** To evaluate the prognostic significance of the eighth edition of the American Joint Committee on Cancer (AJCC) TNM staging classification for gastric cancer.

**Methods:** Prospective databases were reviewed to identify patients who underwent radical gastrectomy at two specialized eastern centers. The prognostic value of the eighth edition TNM classification was estimated and compared with that of the seventh edition. Additional external validation was performed using a dataset from a Western population.

**Results:** Significant differences in 5-year overall survival (OS) rates were observed for each TNM stage when using the eighth edition system, and smaller Akaike information criteria (AIC) values and a higher c-statistic were observed relative to those of the seventh edition. However, the OS rates in each subgroup of stage III patients based on the eighth edition were significantly different. Patients with the same pN stage, namely, the pT4a and pT4b groups, showed similar 5-year OS (P>0.05). Based on the survival data, we propose a simplified staging system. In the improved TNM (iTNM) staging system, the subgroups of a given TNM stage do not show statistically significant differences in OS. The iTNM staging exhibits superior prognostic stratification, with lower AIC values and a higher c-statistic than the eighth edition TNM classification. Similar results were obtained with the external validation dataset from the IMIGASTRIC database.

**Conclusion:** The prognostic prediction of the eighth edition of the AJCC TNM classification is superior to that of the seventh edition. However, it remains associated with some stage migration. The iTNM staging system permits simplification and slightly better prognostic prediction.

## Introduction

Gastric cancer is the third most common cause of cancer-related death 1, 2. Radical resection of the stomach combined with regional lymphadenectomy is the only proven and potentially curative treatment for patients with gastric cancer without distant metastasis 3-5. Several reports on the prognostic implications of the seventh edition of the American Joint Committee on Cancer (AJCC) classification have been published 6-8, and most have found the 5-year survival rates for each seventh edition AJCC stage to differ significantly from each other. Furthermore, they found that the seventh edition classification produced a better prognostic stratification than the sixth edition classification. However, other studies presented conflicting findings 9-11. A European study found that the seventh edition of the AJCC classification was more complex without improving overall survival (OS) prediction in a Western population. They suggested that simplification, with better OS prediction for patients with gastric cancer, should be considered when revising the seventh edition 10. In 2016, the eighth edition of the AJCC/International Union Against Cancer (UICC) TNM staging classification for gastric carcinoma was published 12. This edition retains the same T, N, and M classification as the seventh edition. However, the eighth edition introduced certain changes to the stages, especially for the stage III classification. Therefore, the current study re-evaluated the new AJCC staging system on multi-institutional datasets to ascertain further prognostic implications of the new AJCC classification system. The analysis included direct statistical comparisons of the eighth and seventh edition staging systems and aimed to identify a better TNM classification that would improve prognostic prediction for gastric cancer patients after curative surgery.

## Patients and Methods

### Study population

This study retrospectively analyzed prospective databases at Fujian Medical University Union Hospital (FUUH) and Sun Yat-sen University Cancer Center (SUCC) to identify patients who underwent curative resection (R0) gastric cancer surgery 13. The inclusion criteria were defined as follows: the presence of primary gastric cancer; no preoperative chemotherapy; no distant metastasis; R0 resection (no residual macroscopic or microscopic tumor); more than 15 examined lymph nodes; and records of all relevant values. Patients were excluded if histological findings identified a tumor type other than adenocarcinoma, if the histopathological data were incomplete, if remnant gastric cancer was found, or if either the date of patient death or patient survival data had not been recorded. Patients who died because of postoperative complications were also excluded. A total of 7,191 patients (FUUH: 4,957 vs. SUCC: 2,234) were ultimately included in the study as the development cohort. The inclusive time period of study differed between institutions depending on the availability of data that had previously undergone review by dedicated GI pathologists (FUUH, January 1997 to December 2014; SUCC, January 2000 to December 2012).

All surgical procedures were performed according to the Japanese Research Society for the Study of Gastric Cancer guidelines, including D2 lymphadenectomy 14, 15. Each resected specimen had undergone gross sectioning and histological examination by trained surgical pathologists. The tumor type, local tumor growth, number of lymph nodes resected, and number of lymph node metastases were confirmed histologically. The T classification, N classification, and final staging were all conducted according to both the seventh and eighth editions of the AJCC/UICC TNM classification 16, 12. Adjuvant chemotherapy using 5-fluorouracil (5-FU)-based regimens (mostly oxaliplatin with either Xeloda or S1) was recommended for the majority of patients with advanced gastric cancer 17, 18. Follow-up data were collected from the follow-up office established by the Department of Gastric Surgery or from the Hospital or National Statistical Office data. The survival duration was measured from the time of surgery to either the last date that survival information was collected or to the confirmed date of death. All patients were observed until death or to a final follow-up date of December 2017, whichever occurred first. This study was approved by two local ethics committees. Additional external validation was performed using a Western population dataset from the International Study Group on Minimally Invasive Surgery for GASTRIc Cancer (IMIGASTRIC) trial between 2000 and 2014 with registration number of NCT02325453, which satisfied the aforementioned inclusion and exclusion criteria. Finally, 465 patients were included as a validation cohort.

### Statistical methods

Statistical analyses were performed using SPSS software (version 18.0, SPSS Inc., Chicago, IL) and STATA version 12.0 (StataCorp, College Station, TX). The Kaplan-Meier method was used to estimate time-dependent survival probabilities. Evaluations of monotonicity, distinctiveness, and homogeneity in the respective survival curves were conducted to judge the staging adequacy. The log-rank test was used for statistical comparisons of the survival curves. The relative discriminatory abilities of the different TNM staging systems were assessed using the Akaike information criteria (AIC) and Harrell's concordance index (c-statistic). The general area under the receiver operating characteristic curve quantified the percentage of all patient pairs for whom the predicted and observed survival outcomes were concordant. In general, a predictive model with a low AIC indicates a better model fit, and a high c-statistic represents better discriminatory ability 19-21. Significant differences were assumed at P values of less than 0.05 in a two-tailed test.

## Results

### Clinicopathological characteristics of the patients

In the development cohort, 7,191 patients who underwent radical resection for gastric cancer fulfilled all the inclusion criteria (Supplemental Table 1), including 5,270 (73.3%) males and 1,921 (26.7%) females aged between 12 and 92 years (58.5±11.7 years). The average tumor diameter was 5.1±2.8 cm, and the median number of lymph nodes (LNs) was 27 (range, 15-108). Patients were categorized according to the primary site of gastric cancer: 2,994 (41.6%) had lower-third (L) tumors, 1,318 (18.3%) had middle- third (M) tumors, 2,128 (29.6%) had upper-third (U) tumors, and 751 (10.4%) had tumors located at two or more positions in the stomach. Based on the eighth edition TNM classification 12, 1,167 (16.2%) patients had stage pT1, 831 (11.6%) had stage pT2, 1391 (19.3%) had stage pT3, 3098 (43.1%) had stage pT4a, and 704 (9.8%) had stage pT4b disease. A total of 2,257 (31.4%) patients showed no LN metastasis, and 1064 (14.8%) had pN1, 1,330 (18.5%) had pN2, 1,587 (22.1%) had pN3a, and 953 (13.3%) had pN3b disease. According to the TNM classification, 940 (13.1%) patients were stage IA, 554 (7.7%) were stage IB, and 604 (8.4%) were stage IIA; these values were identical using both the seventh and eighth editions. In the seventh edition, 917 (12.8%) were stage IIB, 787 (10.9%) were stage IIIA, 1,277 (17.8%) were stage IIIB, and 2,112 (29.4%) were stage IIIC. However, in the eighth edition, 913 (12.7%) were stage IIB, 1,506 (20.9%) were stage IIIA, 1,513 (21.0%) were stage IIIB, and 1161 (16.1%) were stage IIIC.

### Long-term surgical outcomes

**Figure 1:**
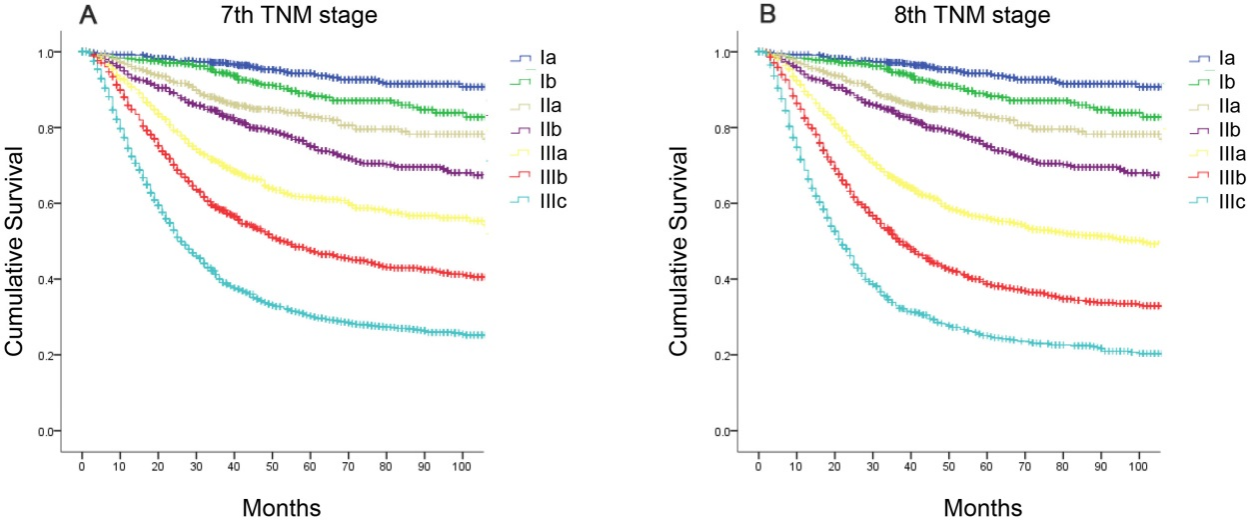
** Comparison of survival curves according to the AJCC TNM staging system. (A)** the seventh edition; **(B)** the eighth edition.

The median follow-up period was 68.0 (range, 1-218) months for the development cohort. The 5-year OS rate of the entire cohort was 59.4%. According to the seventh edition, the 5-year OS rates were as follows: stage IA, 94.4%; stage IB, 88.7%; stage IIA, 82.8%; stage IIB, 75.1%; stage IIIA, 61.6%; stage IIIB, 47.5%; and stage IIIC, 30.3% (Figure 1A, χ^2^=1754.47, P<0.001). According to the eighth edition, the 5-year OS rates were as follows: stage IA, 94.4%; stage IB, 88.7%; stage IIA, 82.8%; stage IIB, 75.1%; stage IIIA, 56.2%; stage IIIB, 38.7%; and stage IIIC, 25.1% (Figure 1B, χ^2^=1866.45, P<0.001).

Statistical analyses of the predictive performance of the two staging systems revealed the superiority of the eighth edition AJCC TNM classification compared with the seventh edition. The eighth edition of the TNM staging system had a smaller AIC value (44313.97 vs 44272.50, respectively) and higher Harrell's c-index (0.736 vs 0.730, respectively), representing the optimum prognostic stratification.

### Survival according to the T category and N category subgroups in the eighth edition of the TNM classification

**Figure 2:**
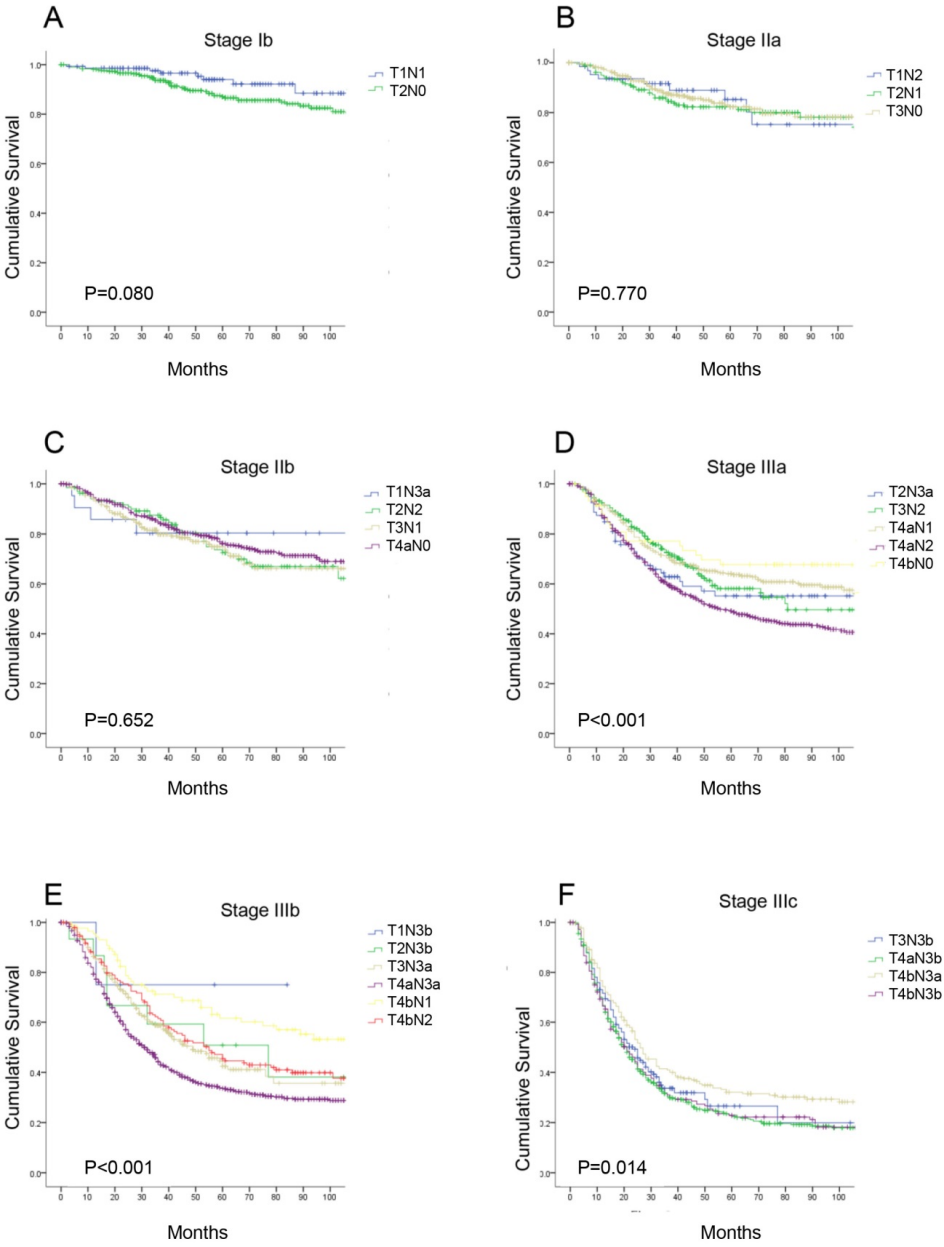
** The survival curves of each subgroup in the same 8^th^ TNM stages. (A)** stage Ib; **(B)** stage IIa; **(C)** stage IIb; **(D)** stage IIIa; **(E)** stage IIIb; **(F)** stage IIIc.

According to the eighth edition TNM classification, there are seven different substages for patients with radical gastrectomy, and each stage includes subgroups with different T or N categories. We performed a detailed analysis comparing the 5-year OS of each subgroup of patients in the same TNM stage (Supplemental Table 2). There were no significant differences in the OS curves in each subgroup at stage IB, IIA, and IIB (P>0.05). However, in stages IIIA, IIIB and IIIC, the 5-year OS in each subgroup were significantly different (P<0.05, Figure 2). Additionally, we found the survival of pT1N3b (75.0%) and pT2N3b (50.8%) patients was better than that of stage IIIb patients, such as those with stage pT3N3a disease (40.0%); and within the same N category, the 5-year OS rate of patients with stage pT4a disease was similar to that of patients with stage pT4b disease (e.g., pT4aN1 vs pT4bN1, 64.0% vs 61.6%, P>0.05, respectively).

### Comparison of the OS between pT4a and pT4b patients in the same N category

**Figure 3:**
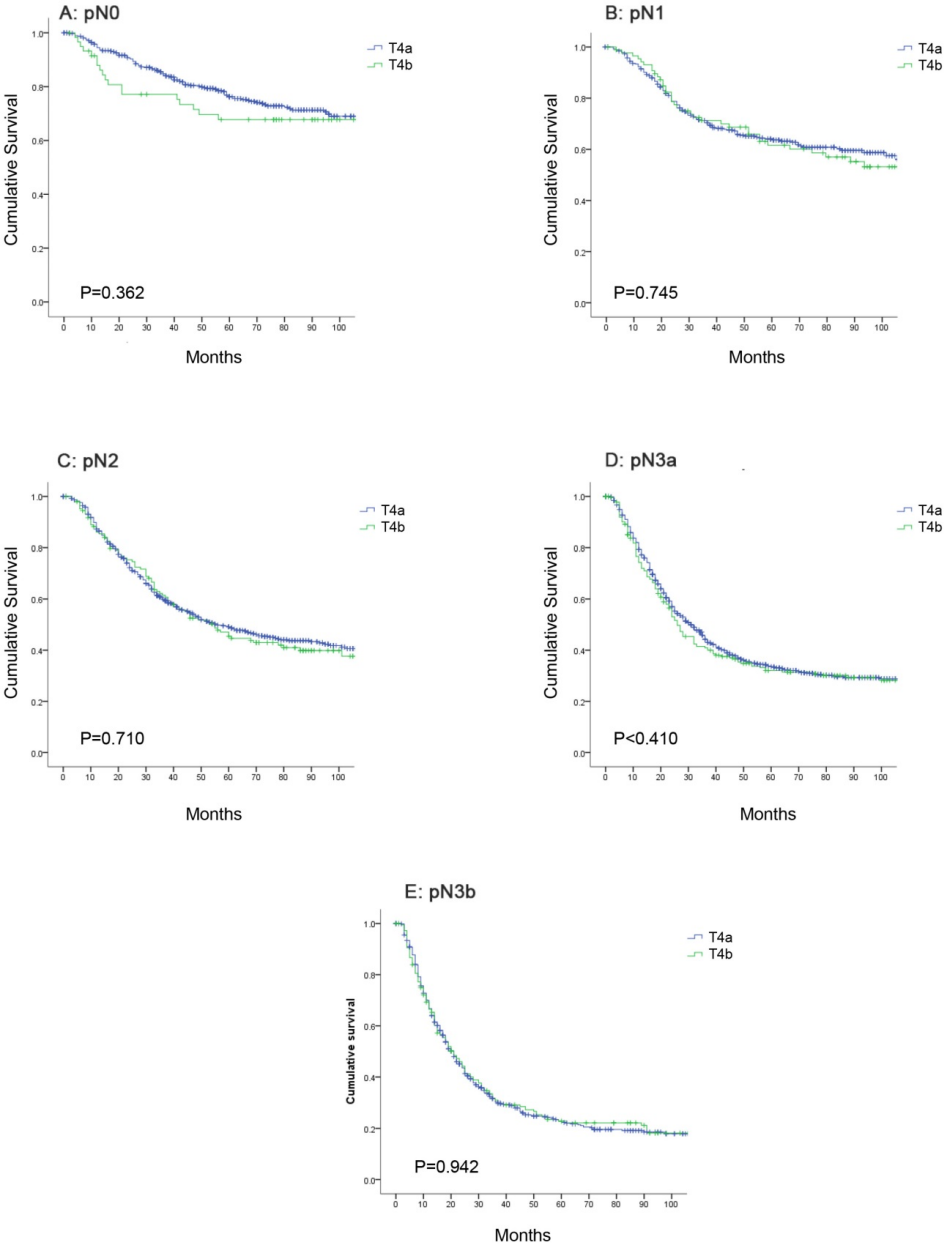
** Comparison of overall survival between pT4a and pT4b patients in the same N category. (A)** pN0; **(B)** pN1; **(C)** pN2; **(D)** pN3a; **(E)** pN3b.

Further analysis indicated that the 5-year OS rates for patients with stage pT4a and pT4b disease in the same N category were similar. The 5-year OS rates for patients with stage pN0 disease was 76.2% in the pT4a group and 67.7% in the pT4b group; for patients with stage pN1 disease, the rates were 64.0% and 61.6%; for patients with stage pN2 disease, the rates were 49.1% and 45.5%; for patients with stage pN3a disease, the rates were 33.6% and 32.0%; and for patients with stage pN3b disease, the rates were 22.9% and 22.8%, respectively. The OS curve did not show any significant differences between stage pT4a and pT4b patients of the same N category (Figure 3, P>0.05).

### Proposal for and survival analysis of an improved TNM staging system

**Figure 4:**
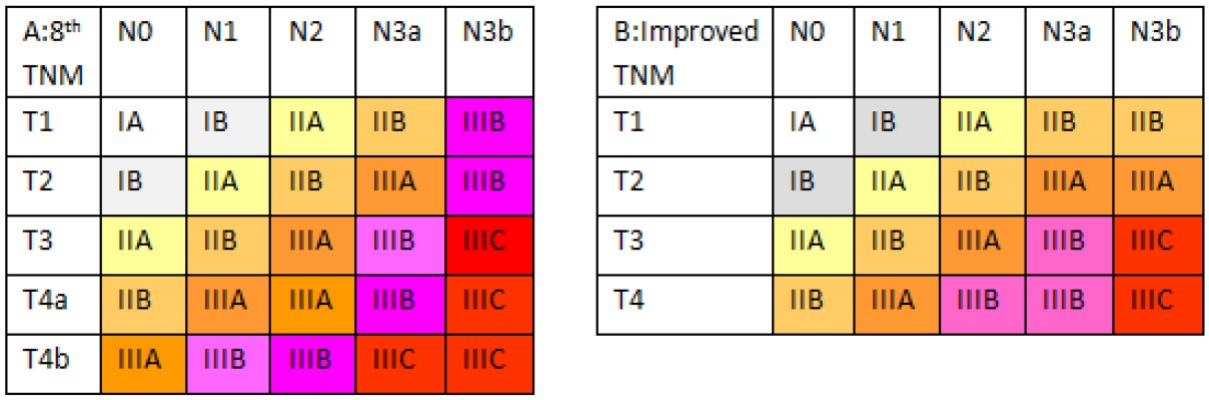
** Differences in the two classifications. (A)** The eighth edition TNM staging system. **(B)** The improved TNM staging system.

Based on these survival data, we revised the eighth edition of the AJCC TNM classification (Figure 4). In the improved TNM (iTNM) staging system, we simplified the pT4a and pT4b subcategories as a single pT4 category. Meanwhile, we retained the pT1-2N3b stages, similar to the seventh edition classification. Kaplan-Meier plots of this improved stage grouping showed statistically significant differences among the individual stage subgroups without any intersecting curves, and the curves appeared more equally distributed (Supplemental Figure 1; χ^2^=1,901.78, P<0.001). In addition, the 5-year OS in each subgroup of the new stages IIB, IIIA, IIIB, and IIIC showed better homogeneity (Table 1).

### Prognostic value of the eighth edition and iTNM staging systems

Statistical assessment of the predictive performance of the two staging systems revealed the superiority of the iTNM classification compared with the eighth edition of the UICC TNM staging system. The iTNM system had a smaller AIC value (44,148.68 vs 44,272.50 for the eighth edition) and a higher Harrell's c-index (0.740 vs 0.736, respectively).

An external validation of the new staging system was performed using a Western dataset (n=465) from the IMIGASTRIC trial (Supplemental Table 1). The median follow-up period was 47.0 (range, 1-176) months. In the validation cohort, the 5-year OS was significantly different between the eighth edition of the AJCC TNM and the iTNM at most stages (P<0.05; Supplemental Figure 2). The iTNM staging also showed a slightly smaller AIC value (1,268.37 vs 1,271.56 for the eighth edition) and a higher Harrell's c-index (0.754 vs. 0.752, respectively), representing a more optimal prognostic stratification than the eighth edition of the AJCC staging classification for gastric carcinoma.

## Discussion

The accuracy of a staging system in predicting long-term survival among patients with gastric cancer is pivotal to help guide postoperative treatment decisions and follow-up 22. Currently, the AJCC system is the most widely utilized staging system; it stratifies M0 gastric cancer into seven risk groups according to the pathological depth of invasion and the number of metastatic LNs. Many studies have reported that the seventh edition system performs better than the sixth edition system in several aspects 23, 24. However, some evidence has demonstrated that the seventh edition TNM staging did not resolve all the problems of previous editions 10, 25. Nodal status is a singularly important prognostic factor in gastric cancer. Until now, the classification of the N stage has been controversial 26, 27. When utilizing the N staging system, more than 15 retrieved LNs are required for optimal staging 28-30. In our study, the median LN retrieval was 27 (range, 15-108), making it adequate for N staging. To provide greater monotonicity, distinctiveness, and homogeneity, the subdivisions of the N classification, which are based on the number of metastatic LNs, were changed in the seventh edition. The N1 substage in the sixth edition system was divided into N1 and N2 in the seventh edition system, and the N2 and N3 substages were merged into the N3a and N3b groups. However, the N3a and N3b groups usually have the same TNM stages in the seventh edition. In the recently revised eighth edition of the TNM staging system of gastric cancers, several important changes were made. The major change in the eighth edition was focused on stage III. The placement of some pN3a patients was revised into stage IIIB, and pT3-4bN3bM0 was classified as stage IIIC 12. Although previous studies have confirmed the prognostic value of the 8th AJCC stage system for gastric cancer (GC), it is mostly confined to patients with stage III 31, lymph node negative or lymph node the 15-Lymph Node Minimum 32, 33. Our study firstly evaluated the prognostic value of the 8th AJCC stage system for resectable GC from multicenter database.

Currently, the 5-year survival rates for patients with radical gastrectomy range between 38.7% and 78.1% 23-25, 34. In the present study, the 5-year OS rate was 59.4%. After reclassifying our patients according to the eighth- and seventh edition TNM staging systems, there were statistically significant differences for each stage, consistent with other reports 35-37. However, the eighth edition of the TNM staging system showed better predictive ability (indicated by a low AIC value and a high c-statistic) for patients, thereby serving as a good staging system to reflect “decreased patient survival with increasing stage group (monotonicity), difference in survival among groups (distinctiveness), and similar survival with in a group (homogeneity)”.

In the stratified analysis, we found that in stages IIIB and IIIC in the eighth edition, the 5-year OS rates in each subgroup were significantly different. This finding revealed that the eighth edition stage stratification of gastric cancer patients does not comply with the general criteria for stage grouping outlined in the introduction of the TNM classification. After carefully observing the data for stage IIIA, IIIB and IIIC, we observed two phenomena. First, the survival rates of pT1N3b (75.0%) and pT2N3b (50.8%) patients were not as inferior as that of stage IIIB patients. Second, within the same N category, the 5-year OS rates of pT4a patients were similar to those of pT4b patients. In some previous studies, the OS rates of pT4a and pT4b patients were significantly different 21, 38. However, those analyses were not based on the same pN category. pT4b patients might have more LN metastases with worse survival, leading to different OS rates from those of pT4a patients. Therefore, we compared the OS rates of pT4a and pT4b patients within the same N categories and found that none were significantly different (P>0.05). After a radical operation, patients with pT4a- or pT4b-stage disease may have similar survival. Therefore, our results indicate that identifying the depth of tumor invasion and classifying T4a and T4b patients with radical gastrectomy are unnecessary.

Based on these survival data, we aimed to revise the stage grouping based on the median patient survival and on the general rules of stage grouping as outlined by the AJCC. The improved stage grouping was as follows (Figure 4). First, the pT4a and pT4b subgroups were combined into the pT4 category. Meanwhile, based on 5-year OS rates, stage IIB was stratified into pT1N3a-3b, pT2N2, pT3N1, and pT4N0 tumors; stage IIIA comprised pT2N3a-3b, pT3N2, and pT4N1 tumors; stage IIIB was redefined as pT3N3a and pT4N2-3a tumors; and stage IIIC was redefined as pT3-4N3b tumors. Stages IA, IB and IIA were identical to those in the eighth edition. The Kaplan-Meier plots of this modified stage grouping were more equally distributed and showed no statistically significant differences among the subgroups in most individual stages and had no crossed curves. Moreover, this iTNM staging system is simpler and has better predictive ability (with a lower AIC value and a higher c-statistic) for patients, revealing the potential superiority of the iTNM classification over the eighth edition of the AJCC TNM staging system.

This study is significant, as a large multi-institutional cohort of patients who underwent radical gastrectomy and had a verified diagnosis based on the latest revision of the AJCC TNM classification was used to develop the iTNM staging system. Before considering whether to use a clinical prediction model, an external validation is essential to ensure its external applicability, although the predictive accuracy may decrease within the external validation set 39, 40. The iTNM staging system in this study was established by the Eastern population, and previous studies have shown that there are significant differences in the incidence of gastric cancer between the East and the West 41, 42. Therefore, additional external validation was performed using a Western dataset. The external validation in this study was performed using a Western dataset from the IMIGASTRIC trial, which was registered at clinical trials.gov with a registration number of NCT02325453. To obtain an actual N category, we included only patients who had more than 15 examined nodes in the validation set from the validation cohorts. Because the incidence of gastric cancer is relatively low in Western countries, only 465 cases were included in the validation set. However, we obtained similar results in the IMIGASTRIC dataset, which further showed that the iTNM stage can better predict the prognosis of GC patients than the eighth edition TNM staging system, and has a certain universality.

There are still several limitations to this study. First, the sample size of the validation set was slightly small, which may limit the power of our conclusions. Second, the results of this study cannot be directly translated to patients who were either treated with inadequate lymphadenectomy or with fewer than 15 retrieved LNs. The iTNM classification might be most valuable and reproducible when combined with a standard and adequate lymphadenectomy and a sufficiently thorough examination of LNs.

## Supplementary Material

Supplementary figures and tables.

## Figures and Tables

**Table 1:** Survival according to the T category and N category subgroup in each iTNM classification

Stage	TN stage	5-year OS(%)	p value-1	p value-2	p value-3	p value-4	p value-5
**IIb**	T1N3a	80.4	-	0.800	0.645	0.604	0.761
	T1N3b	75.0	0.800	-	0.877	0.913	0.801
	T2N2	72.5	0.645	0.877	-	0.789	0.581
	T3N1	74.9	0.604	0.913	0.789	-	0.388
	T4N0	75.3	0.761	0.801	0.581	0.388	-
**IIIa**	T2N3a	55.2	-	0.502	0.405	0.266	
	T2N3b	50.8	0.502	-	0.299	0.168	
	T3N2	58.1	0.405	0.299	-	0.626	
	T4N1	63.5	0.266	0.168	0.626	-	
**IIIb**	T3N3a	42.4	-	0.276	0.062		
	T4N2	48.3	0.207	-	0.001		
	T4N3a	35.3	0.062	0.001	-		
**IIIc**	T3N3b	26.5	-	0.335			
	T4N3b	22.8	0.335	-			

*P: compared with each subgroup in the same TNM stage; p value-1: compared to the first subgroup; p value-2: compared to the second subgroup; p value-3: compared to the third subgroup; p value-4: compared to the fourth subgroup; p value-5: compared to the fifth subgroup
