# Supplementary material for: Multicenter Validation Study of the American Joint Commission on Cancer (8th Edition) for Gastric Cancer: Proposal for a Simplified and Improved TNM Staging System
Source: J Cancer. 2020 Mar 13;11(12):3483–91. doi: 10.7150/jca.36891 (PMC7150461; doi:10.7150/jca.36891)
Supplement: Supplementary file 1 — Supplementary figures and tables. [file jcav11p3483s1.pdf]

Figure Legends

Supplemental Figure 1. Comparison of survival curves according to the iTNM staging system.

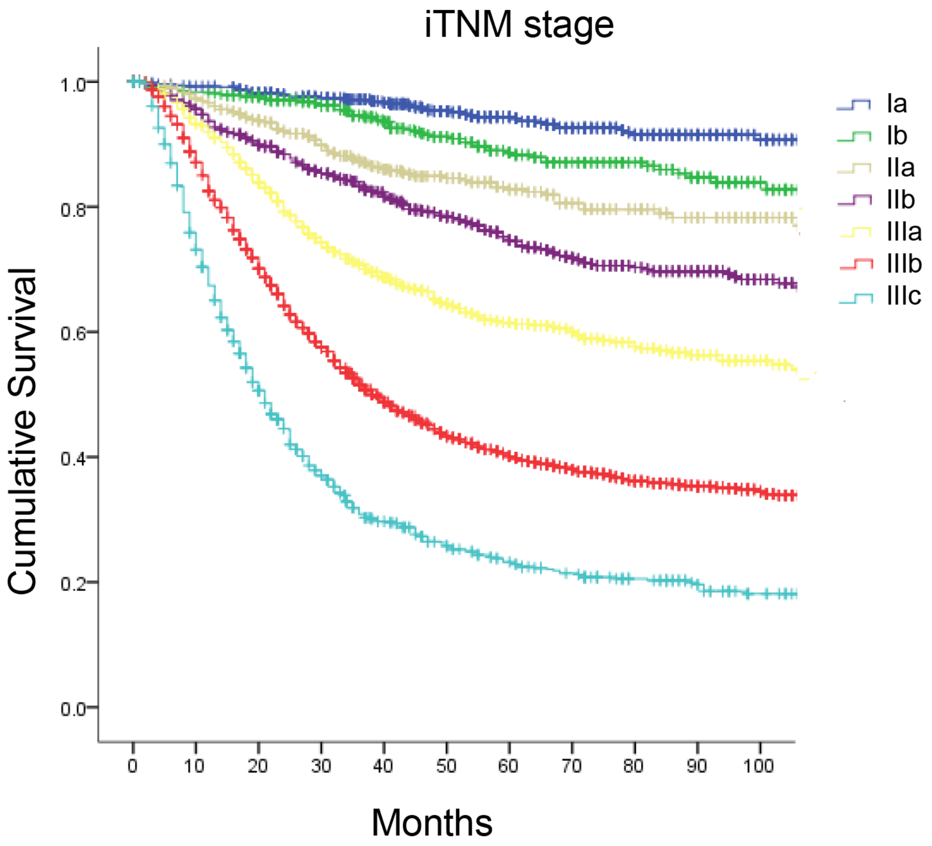

Supplemental Figure 2. The survival curves of the validation set. (A) According to the eighth edition TNM staging system; (B) According to the improved TNM staging system.

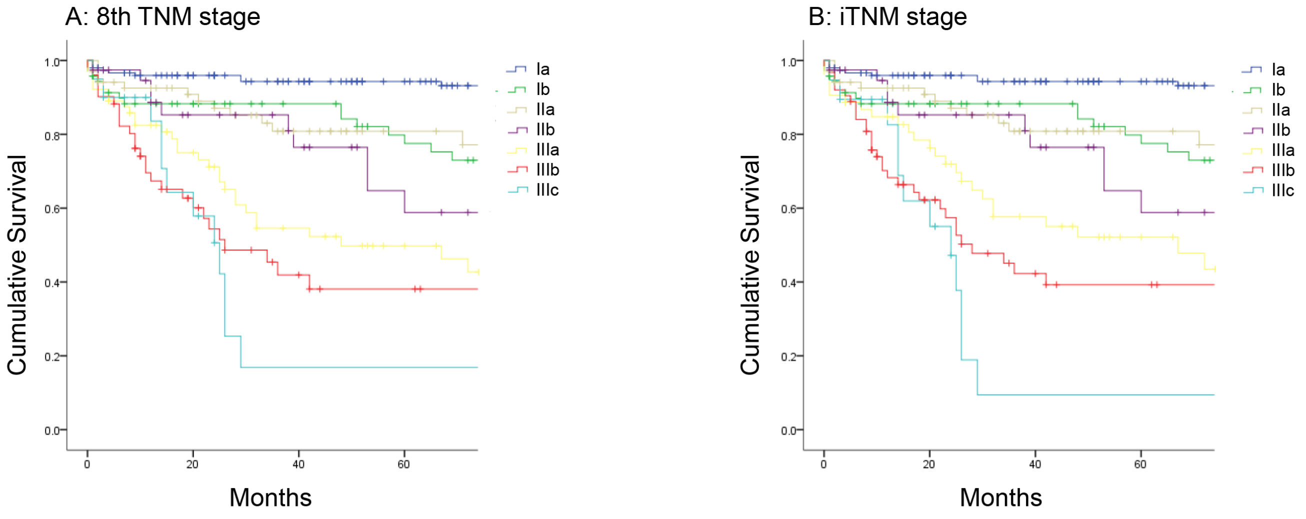

Supplemental Table 1 Clinicopathological characteristics of the patients

Characteristic	Development Cohort N=7191(%)	Validation Cohort N=465(%)
Age (median±SD)	58.5±11.7	67.9±11.2
Sex		
Male	5270(73.3)	270(58.1)
Female	1921(26.7)	195(41.9)
Tumor size (cm, median±SD)	5.1±2.8	4.2±2.4
Tumor location		
Upper	2128(29.6)	109(23.4)
Mid	1318(18.3)	155(33.3)
Lower	2994(41.6)	201(43.2)
Mix	751(10.4)	
Histological type		
Differentiated	1667(23.2)	246(52.9)
Undifferentiated	5524(76.8)	219(47.1)
Type of gastrectomy		
Total	3165(44.0)	225(48.4)
Distal	2733(38.0)	233(50.1)
Proximal	1293(18.0)	7(1.5)
LNs resected; median ±SD	28.4±12.6	27.9±12.2
pT category		
1	1167(16.2)	175(37.6)
2	831(11.6)	116(24.9)
3	1391(19.3)	112(24.1)
4a	3098(43.1)	58(12.5)
4b	704(9.8)	4(0.9)
pN category		
N0	2257(31.4)	241(51.8)
N1	1064(14.8)	82(17.6)
N2	1330(18.5)	56(12.0)
N3a	1587(22.1)	65(14.0)
N3b	953(13.3)	21(4.5)
7th TNM stage		
IA	940(13.1)	151(32.5)
IB	554(7.7)	71(15.3)
IIA	604(8.4)	69(14.8)
IIB	917(12.8)	39(8.4)
IIIA	787(10.9)	53(11.4)
IIIB	1277(17.8)	50(10.8)
IIIC	2112(29.4)	32(6.9)
8th TNM stage		
IA	940(13.1)	151(32.5)
IB	554(7.7)	71(15.3)
IIA	604(8.4)	69(14.8)
IIB	913(12.7)	39(8.4)
IIIA	1506(20.9)	64(13.8)
IIIB	1513(21.0)	51(11.0)
IIIC	1161(16.1)	20(4.3)

Supplemental Table 2 Survival according to the T category and N category subgroups in the eighth edition of the TNM classification

Stage	TN stage	5-year OS(%)	P-1*	P-2*	P-3*	P-4*	P-5*	P-6*
Ia	T1N0	94.4	-					
Ib	T1N1	94.0	-					
	T2N0	87.0	0.080					
IIa	T1N2	85.2	-	0.654	0.963			
	T2N1	82.2	0.654	-	0.468			
	T3N0	82.3	0.963	0.468	-			
IIb	T1N3a	80.4	-	0.654	0.604	0.820		
	T2N2	72.5	0.654	-	0.789	0.483		
	T3N1	74.9	0.604	0.789	-	0.261		
	T4aN0	76.2	0.820	0.483	0.261	-		
IIIa	T2N3a	55.2	-	0.405	0.258	0.283	0.189	
	T3N2	58.1	0.405	-	0.626	0.002	0.169	
	T4aN1	64.0	0.258	0.626	-	0.000	0.388	
	T4aN2	49.1	0.283	0.002	0.000	-	0.005	
	T4bN0	67.7	0.189	0.169	0.388	0.005	-	
IIIb	T1N3b	75.0	-	0.475	0.403	0.243	0.812	0.461
	T2N3b	50.8	0.475	-	0.843	0.322	0.230	0.977
	T3N3a	42.4	0.403	0.843	-	0.000	0.005	0.520
	T4aN3a	36.6	0.243	0.322	0.000	-	0.000	0.001
	T4bN1	61.6	0.812	0.230	0.005	0.000	-	0.031
	T4bN2	45.5	0.461	0.977	0.520	0.001	0.031	-
IIIc	T3N3b	26.5	-	0.340	0.181	0.436		
	T4aN3b	22.9	0.340	-	0.002	0.942		
	T4bN3a	32.0	0.181	0.002	-	0.012		
	T4bN3b	22.8	0.436	0.942	0.012	-		

*P: compared with each subgroup in the same TNM stage; P-1: compared to the first subgroup; P-2: compared to the second subgroup; P-3: compared to the third subgroup; P-4: compared to the fourth subgroup; P-5: compared to the fifth subgroup; P-6: compared to the sixth subgroup
